# Organized immunity in the CNS: what stroke reveals about neuroinflammation and lymphoid niches

**DOI:** 10.1172/JCI202688

**Published:** 2026-03-02

**Authors:** Catalina Lee-Chang

**Affiliations:** 1 Department of Neurological Surgery and; 2 Malnati Brain Tumor Institute, Lurie Cancer Center, Feinberg School of Medicine, Northwestern University, Chicago, Illinois, USA.

## Abstract

Organized adaptive immunity can emerge in the CNS under specific inflammatory and stromal conditions. The study by Yang et al. in this issue of the *JCI* reports that experimental ischemic stroke induced germinal center–like B cell follicles through microglial MIF–CD74/CXCR4 signaling and in situ B cell proliferation, promoting chronic neuroinflammation. These findings align with a growing body of evidence that the brain and meninges can support ectopic lymphoid structures in multiple sclerosis, during aging, and in certain gliomas. This Commentary integrates these observations to highlight shared principles, disease-specific outcomes, and unresolved questions regarding the identity and function of lymphoid aggregates in the CNS.

## Revisiting immune privilege in the CNS

For much of the modern immunology era, the CNS was viewed as an organ fundamentally resistant to adaptive immune organization. This view has softened as studies across neurological diseases have revealed that the brain and meninges can support immune aggregates ranging from simple B cell clusters to partially organized tertiary lymphoid structures (TLSs). These niches vary widely in architecture and function: in some settings, they amplify tissue injury, while in others, they support robust local immune responses (1).

The study by Yang et al. (2) shows that ischemic stroke induced germinal center–like B cell follicles within injured brain parenchyma. Yang et al.’s findings expand the spectrum of conditions in which the CNS can host structured adaptive immune activity and highlight how chronic inflammation can reshape CNS immunity long after the initial insult.

## B cell follicle formation after stroke

**Figure 1:**
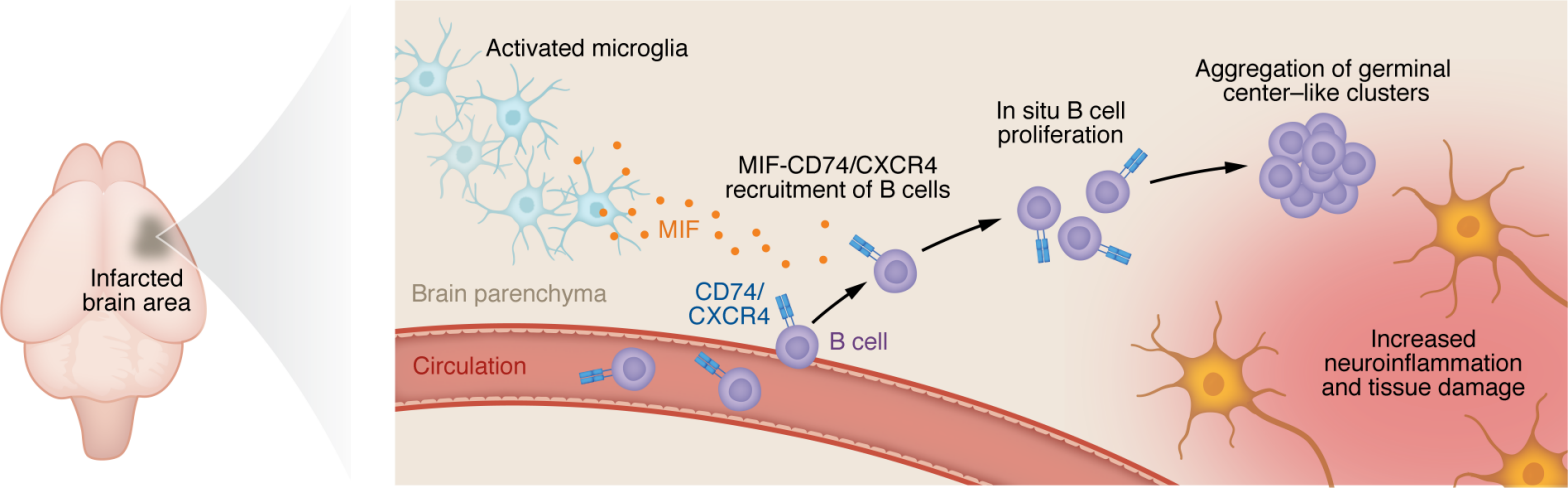
Germinal center–like B cell follicles formed after ischemic stroke exacerbate chronic neuroinflammation and tissue damage. In a mouse model of ischemic stroke, Yang et al. (2) reported that activated microglia released MIF to recruit CD74- and CXCR4-expressing B cells to the site of injury. Recruited B cells underwent in situ proliferation and formed aggregates with features of germinal centers such as activation of IFN-related programs. The presence of germinal center–like B cell aggregates was associated with increases in chronic neuroinflammation and tissue damage. These findings add to growing evidence for organized adaptive immune responses in the brain and a potential role for lymphoid architecture in neuroinflammation and glioblastoma.

Using middle cerebral artery occlusion as a mouse model of ischemic stroke, Yang et al. described a coordinated process driven by microglia secreting macrophage migration inhibitory factor (MIF), which engaged CD74 and CXCR4 on B cells, drawing them into infarcted regions (2). Parabiosis experiments demonstrated that these B cells expanded primarily through in situ proliferation rather than continuous recruitment from the circulation. Over time, the aggregates acquired features reminiscent of germinal centers, including activation markers and IFN-related programs, and their presence worsened chronic neuroinflammation and tissue damage (Figure 1). These results position stroke alongside other conditions in which CNS injury creates an environment permissive to ectopic lymphoid organization.

## TLS-like structures in neurological disease

The stroke-induced follicles described by Yang et al. align with lymphoid structures reported in multiple sclerosis (MS), aging, and neuroinflammation. In MS, meningeal B cell follicles containing proliferating B cells, T cell zones, plasma cells, and stromal networks resembling follicular dendritic cells contribute to cortical demyelination and rapid clinical progression (3, 4). These represent one of the clearest examples of relatively mature TLS-like structures in the CNS.

Aging provides further evidence of meningeal permissiveness to lymphoid aggregation. Fruitman-Davidi et al. recently demonstrated the accumulation of ectopic lymphatic and lymphoid structures in the human dura during aging, accompanied by endothelial and stromal dysregulation and increased lymphocyte density (5). Though these structures lack classical zonation, they reveal that chronic, low-grade inflammation and stromal remodeling in aging dura can foster organized immune aggregates.

Neuroinflammatory states, such as chronic viral infections, meningitis, and traumatic brain injury, also yield perivascular or meningeal lymphoid aggregates with variable degrees of organization. Some lymphoid aggregates express chemokines like CXCL13 and contain dendritic cells, indicating partial alignment with early lymphoid programs, although full germinal center formation is rarely observed (6–8). Together, these findings underscore that the CNS can generate lymphoid niches when inflammation and supportive stromal conditions intersect.

## Glioblastoma: lymphoid organization in a restrictive tumor microenvironment

Against this backdrop, interest has grown in whether glioblastoma supports TLS-like structures. Recent work, including spatial and single-cell profiling (9), identifies lymphoid aggregates in a subset of diffuse gliomas, characterized by B and T cell compartmentalization, clonal B cell expansion, and occasional plasma cell enrichment. Presence of these lymphoid aggregates correlates with more immune-permissive tumor microenvironments and improved survival.

However, several features of these aggregates highlight unresolved questions about their identity. Many lack hallmark features of mature TLSs seen in peripheral tissues, including follicular dendritic cells, high endothelial venules, and distinct germinal center zones (1, 10). The stromal elements needed for classical TLS initiation — particularly coordinated interactions between lymphoid tissue inducer and organizer cells — have not been clearly delineated in glioblastoma (11). Moreover, these aggregates often arise near leptomeningeal surfaces, where the stromal environment differs substantially from that of the tumor parenchyma. This raises the possibility that some structures may reflect meningeal immune niches extending into tumor-adjacent regions rather than tumor-intrinsic lymphoid organization.

These nuances emphasize the need for deeper mechanistic work to determine whether such structures represent CNS-adapted forms of lymphoid organization or distinct variants shaped by the tumor microenvironment. High-resolution stromal mapping, refined TLS criteria (5), and functional studies will be essential to address these questions.

## Insights from cancer and aging: stromal and molecular determinants

Pan-cancer analyses provide broader context for interpreting CNS lymphoid phenomena. Studies profiling thousands of human tumors reveal that TLS-associated gene programs correspond with antigen presentation, immune infiltration, and immunotherapy response, but vary substantially across tissues (12, 13). This highlights the importance of stromal ecology and metabolic constraints in determining whether TLS-like structures can emerge or mature.

The identification of prostaglandin D2 synthase (*PTGDS*) as a TLS-associated gene in glioblastoma links tumor-intrinsic pathways to immune organization, influencing PD-L1 expression, macrophage polarization, and tumor cell behavior (14). Together with age-associated stromal remodeling in the dura (5), these data reinforce a model in which interactions between inflammation, stromal support, and tissue-specific constraints shape lymphoid organization in the CNS.

## A unifying model of CNS lymphoid niches

Across stroke, MS, aging dura, neuroinflammation, and cancer, a consistent theme emerges: the CNS is not inherently incapable of supporting structured lymphoid architecture. Instead, the extent to which lymphoid aggregates form and mature depends on the chronicity of inflammation, the availability of stromal scaffolds, and the tissue environment’s compatibility with organized immunity. Stroke-induced B cell follicles amplify chronic inflammation within necrotic parenchyma; MS follicles reflect highly organized, pathogenic lymphoid microenvironments in the meninges; aging dura exhibits stromal dysregulation that promotes lymphoid clustering; and glioblastoma presents a restrictive microenvironment in which immune aggregates correlate with improved outcomes but require further characterization to establish their identity.

Understanding these distinctions is essential to determining whether CNS lymphoid structures serve as pathogenic drivers or therapeutic opportunities.

## Therapeutic implications and future directions

The pathway identified by Yang et al. (2) suggests opportunities for mitigating long-term inflammation after stroke by targeting microglial MIF–CD74/CXCR4 interactions or downstream B cell IFN programs. In glioblastoma, strategies aimed at promoting beneficial lymphoid organization — through chemokine modulation, vascular normalization, or enhanced dendritic cell recruitment — remain conceptually appealing but depend on clarifying the identity and function of lymphoid structures. Insights from pan-cancer TLS signatures and immune landscape analyses may help identify patients likely to benefit from such interventions.

As the field advances, integrating CNS-specific stromal biology with high-resolution immune profiling will be essential to define when and how ectopic lymphoid structures arise and whether they can be manipulated to therapeutic advantage.

## Funding support

This work is the result of NIH funding, in whole or in part, and is subject to the NIH Public Access Policy. Through acceptance of this federal funding, the NIH has been given a right to make the work publicly available in PubMed Central.

- The National Cancer Institute (R37CA258426, P50CA221747).
- The Cancer Research Institute (CR68036).
- The Chordoma Foundation.
- The Malnati Brain Tumor Institute.

## Version 1 03/02/2026

Electronic publication
